# Consistency over intensity: atorvastatin 40 mg plus ezetimibe 10 mg versus atorvastatin 80 mg in ACS patients: a comparative study of efficacy, tolerability, and cardiovascular outcomes

**DOI:** 10.1186/s43044-025-00708-1

**Published:** 2025-12-12

**Authors:** Ahmad Samir, Kareem Mahmoud, Mohamed Ashraf, Hossam Elhossary, Hesham Salah Eldin Taha

**Affiliations:** 1 Department of Cardiovascular Medicine, Cairo University, Giza, Egypt; 2 Cardiology Department, Cardiac Center Hail, Ha’il, Saudi Arabia

**Keywords:** Statins, Ezetimibe, Statin associated muscle symptoms (SAMS), Acute coronary syndrome (ACS)

## Abstract

**Background:**

Despite guideline recommendations for high-intensity statins in acute coronary syndrome (ACS) patients, real-world data show underutilization and suboptimal low-density lipoprotein cholesterol (LDL-C) target achievement. The statin-associated muscle symptoms (SAMS) is often a major limitation to drug compliance and hence to achieving LDL-C targets.

**Methods:**

In this prospective, open label, randomized controlled trial, 502 ACS patients were randomized 1:1 to atorvastatin 80 mg monotherapy (Group A) versus atorvastatin 40 mg plus ezetimibe 10 mg (Group AE), aiming to compare the efficacy, safety, and tolerability of both treatment arms. Lipid profiles, safety, and clinical outcomes were assessed at baseline, 6 weeks, 6 months, and 12 months. The primary endpoints were LDL-C reduction, SAMS, and major adverse cardiovascular events (MACE).

**Results:**

The mean age of participants was 56 ± 10 years, with 83% males, 55% diabetics, and 77% hypertensives. Baseline characteristics were comparable between groups. Group AE demonstrated superior LDL-C reduction at all timepoints (*p* < 0.01), with greater improvements in high-density lipoprotein cholesterol (HDL-C) and triglycerides (TG). Group AE also showed significantly fewer events of treatment discontinuation (DC) or dose reduction (DR) (OR 0.50; 95% CI 0.31–0.81) and a 25.7% relative risk reduction (RRR) in the composite of MACE compared to Group A. SAMS and non-limiting muscle aches were significantly lower in Group AE (*p* < 0.05), with no cases of rhabdomyolysis reported. Subgroup analysis confirmed consistent benefits of Group AE in both diabetic and non-diabetic patients.

**Conclusion:**

Atorvastatin 40 mg plus ezetimibe 10 mg demonstrated superior LDL-C reduction, improved tolerability, and fewer MACE compared to atorvastatin 80 mg monotherapy in ACS patients. These findings support the use of combination therapy as a viable starting alternative to atorvastatin 80 mg monotherapy following ACS, particularly in patients likely to have statin intolerance.

## Background

After a significant atherosclerotic cardiovascular disease (ASCVD) event, such as an acute coronary syndrome (ACS) or cerebrovascular accident (CVA), clinical guidelines strongly recommend the immediate initiation of high-intensity statin therapy for secondary prevention [1–3]. Statins are favored not only for their ability to reduce low-density lipoprotein cholesterol (LDL-C) but also for their pleotropic benefits, including plaque stabilization and anti-inflammatory effects [4, 5]. Evidence consistently supports a linear relationship between LDL-C reduction and a decline in major adverse cardiovascular events (MACE) [6–8]. Hence, clinical guidelines advocate for at least a 50% reduction in LDL-C levels, targeting levels below 70 mg/dL or below 55 mg/dL as per the American College of Cardiology (ACC) and the European Society of Cardiology (ESC) recommendations, respectively [2, 3]. High-intensity statins, such as atorvastatin 80 mg (or 40 mg) and rosuvastatin 20 to 40 mg, are the primary agents recommended to achieve these targets, with non-statin therapies reserved for cases with statin intolerance or inadequate response [3].

Despite the clear recommendations, a substantial gap exists between guidelines targets and real-world practice. Several registries and studies have confirmed that high-intensity statins are greatly underutilized, a large proportion of ASCVD patients fail to achieve the guidelines targets, and that only one third of patients achieve the targeted LDL-C goals on statin monotherapy [9–12]. These disparities between guidelines recommendations and the real-world data dictate to appropriately identify and address the challenges to achieving targets.

One key challenge is statin intolerance, particularly those related to statin-associated muscle symptoms (SAMS). Although SAMS may be over-reported, it represents a significant barrier to the consistent use of high-intensity statins, with rates of muscle-related complaints reaching up to 30% in some registries [13, 14]. Such events often lead to statin dose reduction or even discontinuation, compromising LDL-C control in these high-risk cohorts. Although the use of most of non-statin LDL-C lowering agents is restricted by their high cost, ezetimibe is a notable exception. Ezetimibe, which inhibits cholesterol absorption, is an affordable and well-tolerated agent with minimal side effects that can provide an additional 20% reduction in LDL-C on top of statin therapy [15, 16]. The IMPROVE-IT trial demonstrated that adding ezetimibe to simvastatin significantly reduced LDL-C levels and lowered MACE by 6.4% [16].

However, it remains unclear whether combining ezetimibe with atorvastatin 40 mg as a standard practice can offer comparable LDL-C reduction with improved tolerability and compliance as opposed to atorvastatin 80 mg monotherapy. This study aims to address this question, evaluating the efficacy, safety, and tolerability of these two strategies in ACS patients.

## Methods

This prospective open label randomized controlled trial recruited 502 ACS patients who met the eligibility criteria. The inclusion criteria were (1) age between 18 and 75 years, (2) ACS presentation within 48 h [including ST-segment elevation myocardial infarction (STEMI) and non-ST-elevation acute coronary syndromes (NSTE-ACS)], and (3) agreement to participate in the study via signing a written informed consent. Exclusion criteria included (1) current consistent use of atorvastatin 80 mg or rosuvastatin ≥ 20 mg, (2) having limitations or contraindications to statin therapy, such as those with objectively proven statin intolerance or rhabdomyolysis on previous exposure, chronic severe liver disease, or proven statins allergy, (3) confirmed diagnosis of familial hypercholesterolemia, or (4) Pregnancy or planning pregnancy in the following 12 months (for females).

Within 48 h of presentation, a computer-generated sequence was used for randomizing eligible patients in a 1:1 fashion. Group “A” received atorvastatin 80 mg monotherapy (single pill), while group “AE” received atorvastatin 40 mg + ezetimibe 10 mg (single-pill combination). Both arms received a single pill regimen to eliminate any impact of patients’ satisfaction and convenience on adherence to the study drug. However, for logistic issues, the patients were not blinded to their randomization.

A baseline fasting lipid profile was assessed within 24 h of ACS presentation. Eligible patients were recruited after the study protocol was explained and obtaining written informed consent for participation and publication of anonymized study results. ACS management adhered to the latest guidelines, incorporating both interventional and pharmacological strategies.

Clinical assessment included a detailed evaluation of risk factors and comorbidities. Body mass index (BMI) was calculated as body weight divided by squared height in Kg/m^2^. As an indicator for obesity-related health risks, waist circumference was measured in centimeters (cm), over bare skin or light clothing, with the patient standing straight, and at end-expiration. The pre-enrollment medical therapy was meticulously reviewed and recorded, particularly if the patient was previously on low- or intermediate intensity statins.

Besides the standard baseline laboratory assessments, High-sensitivity C-reactive protein (hs-CRP), alanine aminotransferase (ALT), and creatine kinase (CK) were measured at baseline and registered. Participants attended clinical follow-ups at 6 weeks, 6 months, and 12 months with repeating of the fasting lipid profile, hs-CRP, ALT and CK. Patients were also instructed to contact the study team anytime throughout the entire study period for reporting any potential drug-related adverse events or any new major clinical event.

The study outcomes aimed to assess the comparative safety, tolerability and efficacy of the 2 study arms. The safety and tolerability were evaluated through the rates of statin-associated adverse effects, particularly SAMS, as well as rates of mandated drug discontinuation (DC) or dose reduction (DR). DR was mandated when the participant reported consistent bothering symptoms with the prescribed regimen and often was rechallenged after couple of weeks if symptoms improve. DC was mandated if the bothering symptoms persisted despite DR, or in cases of severe myositis or rhabdomyolysis. Re-instituting the treatment usually occurred after 2–6 weeks according to the recommended practice [3]. On the other hand, the efficacy was evaluated through the magnitude of reduction in LDL-C from baseline at 6 weeks, 6 months and 1 year, as well as the rates of MACE (including cardiovascular death, myocardial infarction (MI), stroke, urgent unplanned revascularization, or rehospitalization due to ACS or heart failure) up to 12 months of follow-up. Further subgroup analysis was conducted to evaluate any differential impact of the diabetic status on the effects of the study drug.

### Statistical analysis

The sample size was calculated using MedCalc Statistical Software, (MedCalc Software, Ostend, Belgium; https://www.medcalc.org; 2019) with a type 1 error margin of 0.05 and utilizing a study power of 80%. Based on previous registry data showing SAMS rates ranging from 7% to 29% [17–19], the investigators designed the sample size assuming SAMS rate of 20% in atorvastatin 80 mg arm compared to 10% in the atorvastatin 40 mg + ezetimibe combination. Hence, a minimum sample size of 199 patients in each group was required to reject the null hypothesis. The investigators of this study decided a sample size of 502 patients, (251 in each arm) to compensate for potential loss to follow up or missing data.

Collected data were analyzed using the Statistical Package for Social Science (SPSS 26). After anonymization of patient identifiers, categorical variables were represented as frequencies and percentages, and compared between groups through Fisher’s-exact, while continuous variables were subjected to normality testing, then expressed as mean (± standard deviation) or median (25th- and 75th percentiles), then compared between groups through independent samples *t-*tests or Mann-Whitney U test, as appropriate. Overtime comparisons were performed by repeated measures ANOVA or Mc-Nemar. The Relative Risk Reduction (RRR) between the two groups for a specific event was calculated as (event rate in group A– event rate in group AE)/event rate in group A. The odds ratio (OR) expressed the ratio between the odds of event occurrence between the two groups. The significance level was set at a *p*-value of < 0.05.

### Ethical considerations

The study methodology was designed in agreement with the declaration of Helsinki. The study protocol and consent form were reviewed and approved by the institutional research ethics committee and registered as MD3662020. All patients provided informed written consent for participation and publishing of anonymized study results. Data confidentiality was maintained with complete anonymization of the patients’ data, where coded data was available to the steering committee and statisticians, while only treating physicians having access to patient identities.

## Results

This study included 502 eligible ACS patients who were randomly allocated into two equal groups. Group A received atorvastatin 80 mg monotherapy, while group AE received atorvastatin 40 mg plus ezetimibe 10 mg in a single-pill combination. Apart from statin therapy, OMT and similar secondary prevention instructions (risk factor management, diet, exercise and weight management) were delivered equally to all patients.

**Fig. 1:**
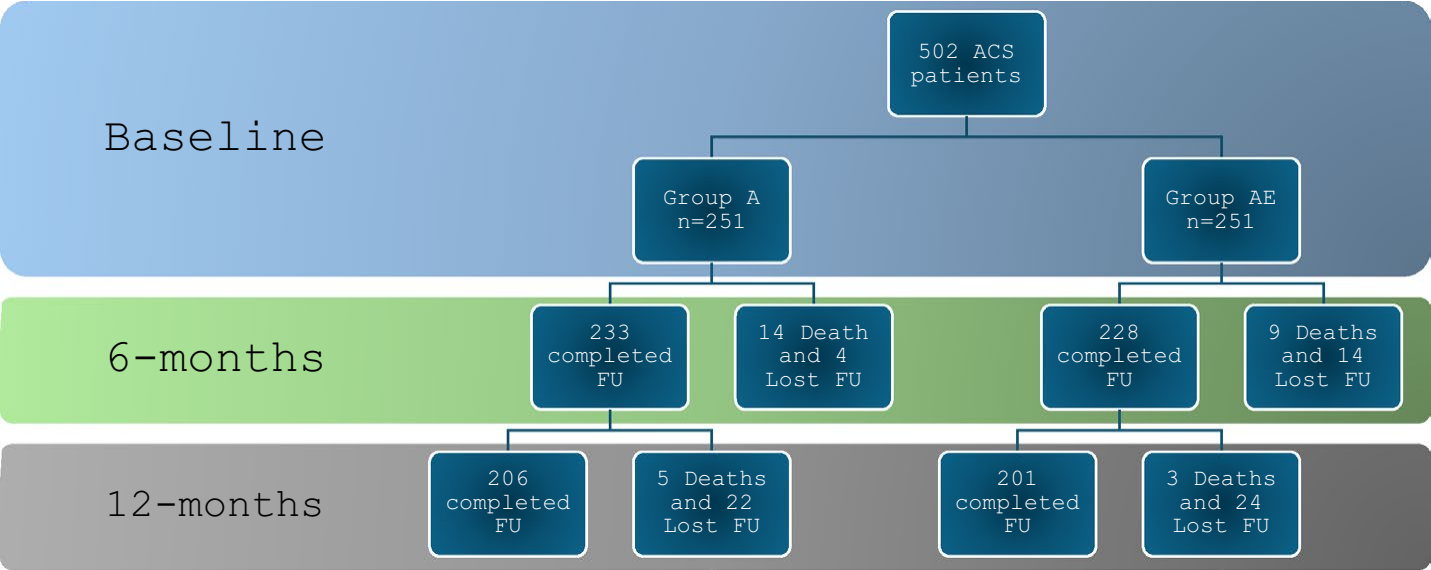
Flow chart for the process of study recruitment and follow-up

The mean age (± SD) of the study group was 56 ± 10 years. Among the recruited cohort, 83% were males, 68% were smokers, 55% had DM, and 77% had hypertension. The presentation of the recruited patients was nearly split between STEMI and NSTE-ACS as 45% and 55%, respectively. The pre-event medical therapy has shown a small and a comparable proportion of using low-or-intermediate statin therapy between the two groups. Also, the baseline lipid profile was comparable between the two study arms. The baseline data of the whole study cohort, as well as the comparative statistics between the study arms are presented in Table 1, while the temporal changes in the fasting lipid profile through the study checkpoints are shown in Table 2. Figure 1 illustrates the randomization process and flow chart for the patients who completed follow-up to the end of the study period.

**Table 1:** Description of the baseline data of the whole study group and comparative statistics between the 2 study arms

	Whole study group (*N* = 502)	Group (A) atorvastatin 80 mg (*N* = 251)	Group (AE) atorvastatin/ezetimibe 40/10 mg (*N* = 251)	*P*- value^*^
Age	58 ± 10	57 ± 9.67	60 ± 10.79	0.862
Male sex	424 (83%)	207 (82.47%)	217 (86%)	0.218
Diabetes mellitus	277(55%)	159 (63%)	118 (47%)	**< 0.001**
Hypertension	388(77%)	195 (78%)	193 (77%)	0.831
Smoker	343 (68%)	168 (67%)	175 (70%)	0.127
BMI (Kg/m^2^)	31.9 ± 2.53	31.6 ± 2.28	32.21 ± 2.72	0.061
Waist circumference (cm)	98.78 ± 7.21	99.9 ± 6.99	97.67 ± 7.26	0.11
ACS present STEMI NSTEMI UA	226 (45%) 167 (33%) 109 (22%)	116 (46%) 84 (34%) 51 (20%)	110 (44%) 83 (33%) 58 (23%)	0.71
Pre-event treatments				
Aspirin	54 (11%)	31 (12%)	23 (9%)	0.94
Clopidogrel	19 (4%)	8 (3%)	11 (4%)	0.98
Ticagrelor	7 (0.01%)	5 (0.02%)	2 (0.01%)	0.122
Statins	82 (16.3%)	42 (16.73%)	40 (15.94%)	0.81
Low-intensity *	48 (58.5%)	24 (57.14%)	24 (60%)	
Intermediate-intensity ^$^	34 (41.5%)	18 (42.86%)	16 (40%)	
Beta-blockers	388 (77.3%)	195 (77.69%)	193 (76.89%)	0.831
Insulin	33 (6.6%)	16 (6.37%)	17 (6.77%)	0.857
Oral-hypoglycemics	273 (54.4%)	157 (62.55%)	116 (46.22%)	**< 0.001**
Baseline laboratory results				
Serum creatinine (mg/dl)	1.41 ± 0.495	1.39 ± 0.49	1.44 ± 0.50	0.239
Haemoglobin (gm/dl)	13.8 ± 1.83	14.3 ± 1.92	13.6 ± 1.74	0.076
First troponin (ng/ml)	12.07 ± 4.03	13.12 ± 3.91	10.02 ± 4.15	0.799
Post-PCI-24 h troponin (ng/ml)	23.25 ± 8.27	20.40 ± 9.21	24.10 ± 8.34	0.686

* Comparative statistics between the (A) and the (AE) groups

ACS: Acute coronary syndrome; BMI: Body mass index; NSTEMI: Non-ST elevation myocardial infarction; PCI: percutaneous coronary intervention; STEMI : ST elevation myocardial infarction; UA: Unstable angina

**Table 2:** Description of lipid profile of whole sample at baseline, 6 weeks, 6 months and one year

	Whole study group	Group (A)	Group (AE)	*P*-value
* At baseline				
Total cholesterol (mg/dl)	190 (± 55.49)	190.42 ± 55.22	191 ± 73	0.632
LDL-C (mg/dl)	124.46 (± 28.74)	124.8 ± 20.55	124.54 ± 25.85	0.952
HDL-C (mg/dl)	37.35 (± 10.8)	36.43 ± 13.3	38.29 ± 7.5	0.054
Triglycerides (mg/dl)	144.41 (± 28.78)	146.58 ± 32.78	142.25 ± 23.98	0.092
* At 6 weeks				
Total cholesterol (mg/dl)	140.5 ± 39.1	142 ± 41.49	138 ± 50.23	0.042
LDL-C (mg/dl)	76.46 ± 46.8	80 ± 38.8	73.24 ± 34	0.001
HDL-C (mg/dl)	38.17 ± 10.85	36.9 ± 13.4	39.5 ± 7.24	0.041
Triglycerides (mg/dl)	93.90 ± 31.66	105.54 ± 34.08	82.25 ± 23.98	0.016
* At 6 months				
Total cholesterol (mg/dl)	152 ± 43.7	171.24 ± 3.74	133.56 ± 45.36	0.335
LDL-C (mg/dl)	91.58 ± 50	112 ± 47.2	71 ± 40.9	0.011
HDL-C (mg/dl)	36.8 ± 12.8	35.6 ± 14.8	38 ± 10.24	0.008
Triglycerides (mg/dl)	93.11 ± 28.14	90.3 ± 27.66	95.91 ± 28.4	0.028
* At 12 months				
Total cholesterol (mg/dl)	139.58 (± 22)	153.3 ± 18.3	125.7 ± 111	< 0.001
LDL-C (mg/dl)	78.56 (± 30)	91.9 ± 46	65.2 ± 33.2	< 0.001
HDL-C (mg/dl)	40.9 (± 11.6)	39.5 ± 14.3	42.2 ± 7.75	0.001
Triglycerides (mg/dl)	88.01 (± 30.56)	97.36 ± 33.41	78.43 ± 23.86	< 0.001

Comparative statistics between serial analyses evaluated by repeated measures ANOVA HDL-C: High-density lipoprotein cholesterol; LDL-C: Low-density lipoprotein cholesterol

**Fig. 2:**
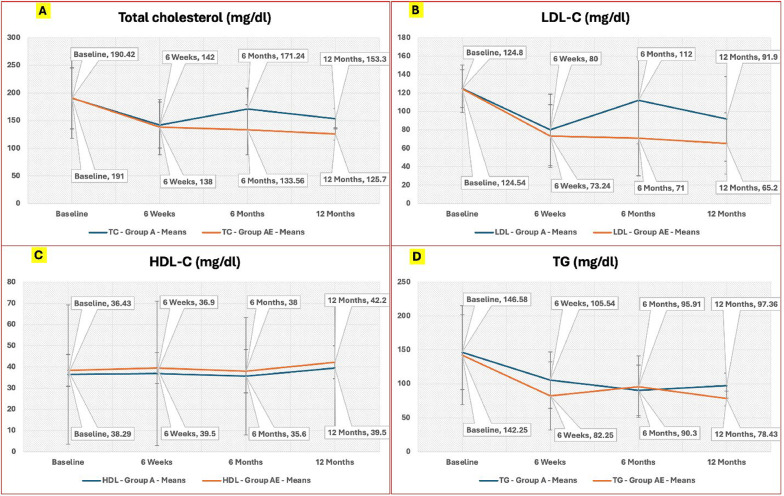
Temporal trends for the lipid profile parameters at baseline and study checkpoints. The values of the fasting lipid profile parameters are represented as mean (callouts over the line-plots) and standard deviation (vertical error bars). **A**, **B**, **C** and **D** represents the total cholesterol, the LDL-C, the HDL-C and the triglycerides (TG) trends, respectively

**Fig. 3:**
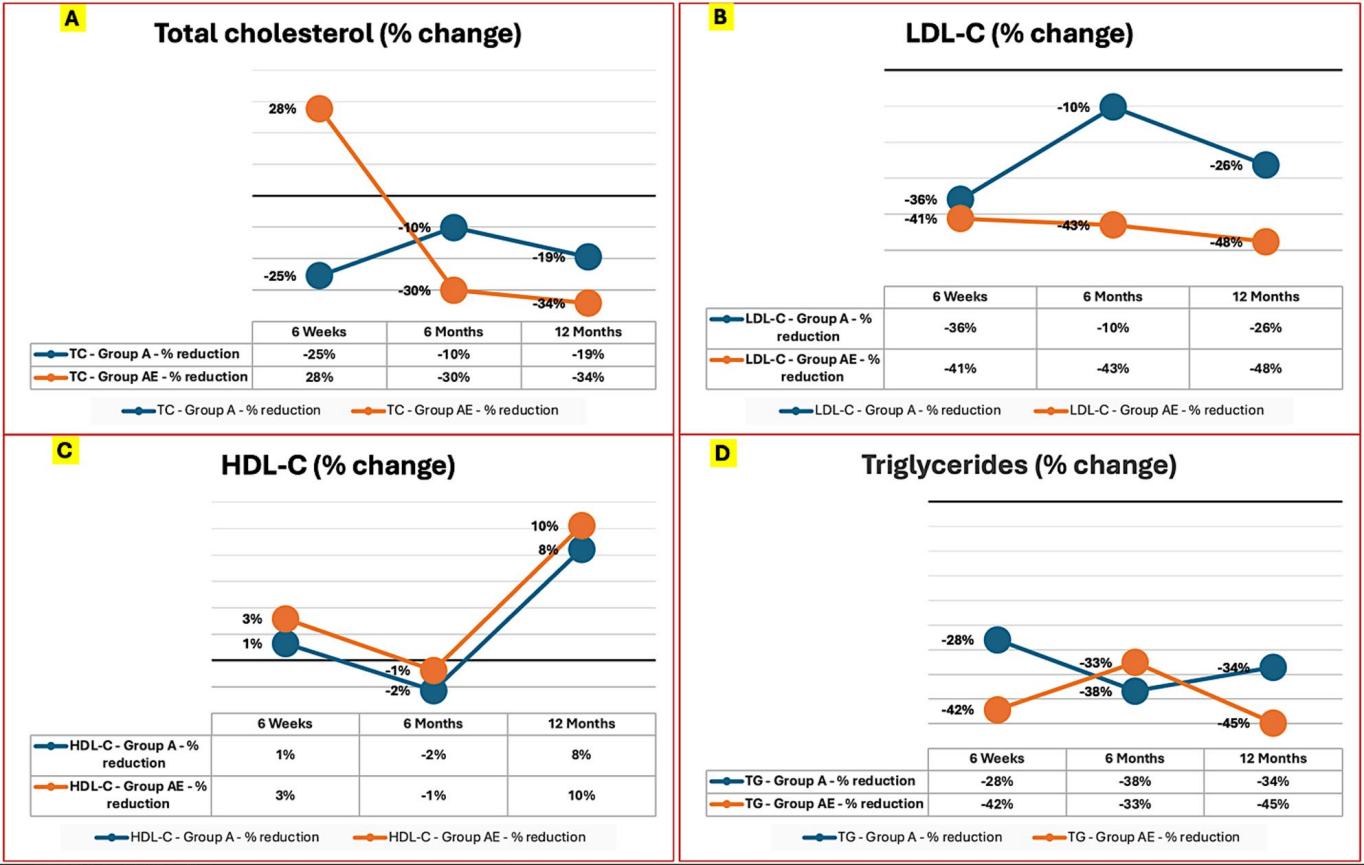
Percent change of the lipid profile parameters at the study checkpoints compared to baseline. **A**, **B**, **C** and **D** represents the serial percent change from baseline for total cholesterol, the LDL-C, the HDL-C and the triglycerides, respectively

Although the lipid profiles were comparable between the two groups at baseline, significant differences started to emerge at the 6-week mark in favour of group AE, who demonstrated higher HDL-C levels and lower LDL-C and triglyceride levels compared to group A. The percent reduction in LDL-C, TG, and TC as well as the improvement in HDL-C levels were more pronounced in group AE at both the 6-month and 12-month follow-ups compared to group A. Table 2; Figs. 2 and 3.

Through the whole study period, group AE demonstrated fewer events of transient DC or DR of the study drug, which were statistically significant for the composite and individual number of events as demonstrated in Table 3. Compared to group A, group AE demonstrated significantly lower odds of treatment DC (OR = 0.51; 95% CI: 0.31–0.83), DR (OR = 0.35; 95% CI: 0.17–0.74), and the composite of DR or DC (OR = 0.50; 95% CI: 0.31–0.81). Also, the results indicated a relative risk reduction (RRR) of 41% for DC, 62% for DR, and 42% for the composite outcome, for group AE compared to group A, highlighting the improved tolerability for statin therapy in group AE.

**Table 3:** Rates of mandated transient dis-continuations or dose reductions of the study drug

	Whole study group	Group (A)	Group (AE)	*P*-value
Dose reduction	88 (22%)	56 (27%)	33 (16%)	0.041
Discontinuation	37 (9%)	27 (13%)	10 (5%)	0.037
Composite of DR-DC	91 (22)	59 (29%)	33 (16%)	0.031

DC: Discontinuation; DR: Dose-reduction

Regarding the clinical endpoints at the end of the study, group AE demonstrated significant reduction in the composite of 6-point MACE (CV death, MI, stroke, urgent unplanned revascularization, and rehospitalization for new ACS or acute HF). The RRR for MACE in the group AE was 25.7% compared to group A. Concerning the individual components of MACE, there was only a trend for reduction of MI (8.8 vs. 17.5%, *p* = 0.080) and subsequent heart failure (7.8% vs. 14.1%, *p* = 0.072), while with non-significant differences in the rates of CV-death, stroke, urgent revascularization in the AE compared to the A group. There was a trend toward a reduction of newly diagnosed DM in group AE compared to group A, occurring at rates of 0.8% and 1.2%, respectively, (*p* = 0.068). Moreover, group AE showed significantly less events of reported bothering SAMS and of non-limiting muscle aches compared to group A. Myositis (defined as muscle pains associated with elevation of the CK for > 4-folds the reference limit) occurred at relatively low rates in both groups with trend towards a lower rate in group AE. There were no reported rhabdomyolysis cases in this study. Table 4.

**Table 4:** Description of adverse events through the 12-month follow-up period

	Whole study group	Group (A)	Group (AE)	*P*-value
MACE	115 (28.2%)	68 (33.1%)	49 (24.6%)	0.048
Cardiovascular death^*^	31 (7%)	19 (8%)	12 (6%)	0.12
MI	53 (13%)	36 (17.5%)	18 (8.8%)	0.08
TIA or stroke	34 (8.4%)	17 (8.1%)	19 (9.3%)	0.15
Urgent unplanned revascularization	77 (19%)	45 (22%)	32 (16%)	0.11
Heart Failure Manifestations	46 (11.2%)	29 (14.1%)	16 (7.8%)	0.072
Newly diagnosed DM	4 (1%)	2 (1.2%)	2 (0.8%)	0.068
Bothering SAMS	63 (15.5%)	39 (19%)	24 (12%)	0.027
Non-limiting muscle aches	168 (41.2%)	119 (57.6%)	50 (25%)	0.006
Myositis	42 (10.4%)	26 (12.7%)	15 (7.3%)	0.059

*Cardiovascular death was counted from the 438 participants (after exclusion of those lost from follow-up), while other endpoints were calculated after exclusion of both deaths and lost follow up, (206 and 201 in group A and group AE, respectively)

DM: Diabetes mellitus; MACE: Major adverse cardiovascular events; MI: Myocardial infarction; SAMS: Statins associated muscle symptoms; TIA: Transient ischaemic attack

### Subgroup analysis by diabetes status

As per study protocol, a dedicated analysis was conducted to illustrate whether a differential impact of the study drug is related to the diabetic status. Hence, the 407 patients who completed the 12-month follow-up were subdivided into diabetics and non-diabetics. Subsequently, the serial changes in LDL-C values were contrasted as per the treatment arm allocation. Basically, diabetic patients had significantly higher LDL-C values compared to the non-diabetic opponents. The magnitude of reduction (response to therapy) demonstrated a significant superiority in favor of AE compared to the A treatment arms across all study checkpoints. This was confirmed by the post-hoc repeated measures ANOVA testing as demonstrated in Table 5. Despite the improved LDL-C reduction, there were no significant differences in the rates of MACE between the 2 treatment arms when stratified by the diabetic status, as shown in Table 6. A multivariable logistic regression model was conducted to assess the impact of treatment allocation (AE vs. A) and diabetes status (Yes vs. No) on MACE occurrence. Diabetes was significantly associated with a higher risk of MACE (OR = 5.79, 95% CI: 3.63–9.24, *p* < 0.001), where diabetic patients had nearly a six-fold increased risk of MACE compared to non-diabetic patients. However, the treatment allocation (AE vs. A) was not associated with significant differences in MACE (OR = 0.92, 95% CI: 0.57–1.47, *p* = 0.717), indicating that the treatment strategy did not independently affect MACE occurrence after adjustment for diabetes. Furthermore, the interaction term (Treatment × Diabetes) was not statistically significant, suggesting that diabetes did not modify the effect of treatment allocation on MACE occurrence.

**Table 5:** Serial LDL-C changes stratified by diabetes status and treatment arm allocation

Parameter	Non-Diabetic (*N* = 140)	Diabetic (*N* = 267)	*P*-value
• Baseline LDL-C (mg/dL)			
Whole group	116.8 ± 22.8	130.6 ± 22.35	< 0.001
Group A (atorvastatin 80 mg)	115.7 ± 26.67	129.31 ± 24.5	< 0.001
Group AE (atorvastatin 40/ezetimibe 10 mg)	117.5 ± 19.9	132.52 ± 19	< 0.001
6 weeks LDL-C (mg/dL)			
Whole group	69.67 ± 18.26	81.97 ± 18.8	< 0.001
Percent change (whole group)	−40.40%	−37.20%	0.22
Group A (atorvastatin 80 mg)	72 ± 21.5	84 ± 20.7	< 0.001
Percent change (group A)	−37.80%	−35.00%	0.004
Group AE (atorvastatin 40/ezetimibe 10 mg)	68 ± 15.5	79.1 ± 15.2	0.001
Percent change (group AE)	−42.10%	−40.30%	< 0.001
6 months LDL-C (mg/dL)			
Whole group	83.93 ± 31.57	97.69 ± 23.82	0.006
Percent change (whole group)	−28.10%	−25.20%	0.22
Group A (atorvastatin 80 mg)	111.5 ± 29	111.6 ± 19.3	0.591
Percent change (group A)	−3.60%	−13.70%	0.004
Group AE (atorvastatin 40/ezetimibe 10 mg)	64.8 ± 14.4	78.7 ± 14.5	< 0.001
Percent change (group AE)	−44.80%	−40.60%	< 0.001
12 months LDL-C (mg/dL)			
Whole group	77.9 ± 24.8	78.88 ± 19.35	0.674
Percent change (whole group)	−33.30%	−39.60%	0.22
Group A (atorvastatin 80 mg)	94.8 ± 30.6	90.29 ± 17.3	0.351
Percent change (group A)	−18.00%	−30.20%	0.004
Group AE (atorvastatin 40/ezetimibe 10 mg)	67.3 ± 11.28	63.57 ± 6.8	0.004
Percent change (group AE)	−42.70%	−52.10%	< 0.001
Repeated measures ANOVA (P-value)			
Whole group	< 0.001*	< 0.001**	
Group A (atorvastatin 80 mg)	< 0.001*	< 0.001*	
Group AE (atorvastatin 40/ezetimibe 10 mg)	< 0.001*	< 0.001**	

*Post-hoc analysis was significant between all follow-ups except between 6 weeks and 6 months in non-diabetic patients (whole group and Group AE)

**Post-hoc analysis was significant between all follow-ups

**Table 6:** Rate of 1-year MACE stratified by diabetic status and treatment arm

	Non-Diabetic (*N* = 140)	Diabetic (*N* = 267)	*P*-value^**^
Whole group MACE	41 (29%)	74 (28%)	0.32
Group A (atorvastatin 80 mg)	27 (19%)^*^	41 (15%)^*^	0.434
Group AE (atorvastatin 40/ezetimibe 10 mg)	14 (10%)^*^	33 (12%)^*^	0.293

^*^Percentages representing rate of occurrence MACE compared to the total of the corresponding group (Non-diabetic or Diabetic)

^**^ Chi-Square by layered cross-tabs contingency tables

## Discussion

This is a prospective randomized controlled study that aimed to compare the tolerability, safety and efficacy of combined atorvastatin 40 mg plus ezetimibe 10 mg opposed to the maximum-atorvastatin dose (80 mg). The study aimed to underscore the importance of consistency of the prescribed strategy for LDL-C lowering, highlighting the need to recognize and overcome the challenges that lead to frequent DR or DC.

This study recruited 502 eligible ACS patients and randomized them as 1:1 into the treatment strategies. The mean age of the study group was 58 years (± 10) which is, as often seen in Middle East, younger than the mean age of ACS patients often encountered in the Western registries [20–22]. Such younger age in our population accentuates the need for intensified secondary prevention for the longer expected lifetime risk.

Undoubtedly, following an ACS, high-intensity statins are cornerstone therapies, for their well-recognized efficacious LDL-C reduction as well as their broad pleotropic cardiovascular benefits [2, 3, 23]. The Cholesterol Treatment Trialists (CTT) collaboration, which analyzed data from over 170,000 patients, established a clear relationship between LDL-C reduction and decreased incidence of MACE, with every 1 mmol/L (38.8 mg/dL) reduction in LDL-C corresponds to a 22% reduction in MACE [24]. Thus, most practice guidelines strongly advocate for a minimum 50% reduction in LDL-C after a major ASCVD event and aiming for levels below 70 mg/dL or 55 mg/dL, in the American College of Cardiology (ACC) or the European Society of Cardiology (ESC) recommendations, respectively [2, 3, 7, 25]. As it is presumed that high-intensity statins (atorvastatin 80 mg (or 40 mg) and rosuvastatin (20-to-40 mg) can reduce LDL-C by ≥ 50% from baseline [2], the guidelines recommended them as monotherapy, sparing the addition of non-statin therapies only for cases failing to tolerate them or to achieve targets in subsequent checks [2, 3].

However, a huge gap was encountered between the real-world data and the guidelines’ recommended targets. For instance, in the National Cardiovascular Data Registry “PINNACLE Registry” that included more than 2.5 million ASCVD patients, 72% were not achieving the LDL-C < 70 mg/dl target, with younger patients (18–64 years) less likely to achieve targets compared to older patients [10]. The same trend was encountered in the “National Health and Nutrition Examination Survey, (NHANES) registry”, which assessed >1600 participants indicated for high-intensity statins [26]. More than two thirds of the recruited ASCVD were not on any statin, while among the group who were on regular statins, 79.7% were not achieving the recommended targets [26]. Further evidence of the substantial gap between the guidelines’ recommendations and real-world data was demonstrated by the results of the EUROASPIRE Registry and the GOULD Registry (Getting to an ImprOved Understanding of Low-Density Lipoprotein-Cholesterol and Dyslipidemia Management) [9, 12].

Furthermore, in the landmark “ISCHEMIA trial” which recruited ASCVD patients with symptomatic and documented occlusive CAD [27], 95% of the recruited patients were on statins at baseline yet, only 41% were receiving high-intensity therapy. More surprisingly, this 41% proportion increased to only 66% by study completion, with only 59% achieving the LDL-C targets in the final report of the study that focused on providing optimal medical therapy (OMT) and demonstrating the noninferiority of OMT compared to PCI [27].

Thereby, the need to identify and address the barriers to consistent utilization of high-intensity statins and achieving of LDL-C targets in real-world clinical practice was sensibly commanded. Statins intolerance due to adverse effects can be one significant barrier that needs to be appropriately scrutinized. Although severe side effects like myositis or rhabdomyolysis are quite rare, SAMS can occur in over 30% of patients on statins therapy and obviously can impact treatment adherence [13, 18, 19]. More importantly, the frequency of SAMS is linearly correlated with the utilized statin dose, thus more likely to complicate the use of high-intensity statins. The evidence that statins-related adverse effects are dose dependent has been confirmed in several large landmark studies and registries. In the large “PRIMO” study that recruited 7924 patients on statin therapy, rates of bothering muscle symptoms occurred in 10.5%, with significantly higher event rates in those receiving higher statins intensity [28]. Further characterization of the prevalence and features of statins intolerance were well reviewed in the International Lipid Expert Panel, the Canadian Consensus Working Group, and the National Lipid Association consensus documents [17–19]. These consensus documents and statements acknowledged statins intolerance, primarily driven by SAMS, as a major hindrance for achieving the recommended LDL-C targets in ASCVD patients in real-world practice.

On the other hand, Ezetimibe which is an inhibitor for dietary cholesterol absorption through the Niemann-Pick C1-Like 1 (NPC1L1) Protein, has shown very well tolerability profile with scarcely reported adverse effects [15]. Almost a decade ago, the IMPROVE-IT trial previously demonstrated that adding ezetimibe to simvastatin reduced LDL-C and MACE in >18,000 ACS patients over 7 years [29, 30]. Nevertheless, the contemporary management of ACS had been revolutionized from the era of the IMPROVE-IT study, with the introduction of potent antiplatelet agents, new generations of drug-eluting stents, and even recognition that Simvastatin is not among the statins that qualify high intensity LDL-C reduction [2].

Hence, the present study builds on these facts by comparing ezetimibe plus atorvastatin 40 mg opposed to atorvastatin 80 mg, ensuring both study arms equally receive the state-of-art interventional- and medical management, including a backbone of high-intensity statins therapy. In this prospective study, group AE demonstrated improved LDL-C reduction through the serial assessments (efficacy), with fewer adverse effects (safety), and significantly less events of DR/DC (tolerability), compared to group A.

For better and full interpretation of these encountered results, we broke the findings into blocks. Firstly, it was very clear that the treatment arm receiving the larger dose of atorvastatin was associated with more of the statins associated side effects, with statistically significant difference in SAMS and non-limiting muscle aches, while with a trend for higher myositis and new DM rates.

Secondly, there is an important argumental point in the elucidation of the encountered magnitude of LDL-C reduction from either treatment arms. Considering baseline therapy with atorvastatin 40 mg, we have learnt from the “Treatment to New Targets, the TNT Study” that uptitration to 80 mg is paralleled by additional 6–12% reduction in the LDL-C levels [31]. On the other hand, addition of ezetimibe is expected to lead to 15–20% extra LDL-C reduction over the background of statin therapy [29, 32]. Thus, from both the theoretical basis and what was confirmed in the results of this study, the magnitude of LDL-C reduction expected from atorvastatin 40 mg + ezetimibe 10 mg can be equivalent or slightly superior to that of atorvastatin 80 mg alone.

While these basic pharmacological concepts seem very rational, they do not reflect the whole truth. Through the study period, it was quite clear that the mandated DR, DC, and their composite, were more frequent in group A compared to group AE, occurring at rates of 27% vs. 16%, 13% vs. 5%, and 29% vs. 16%, respectively. Reasonably, the more frequent DR/DC events in group A were related to the more frequent encounter of bothering muscle symptoms. These more reported DR/DC undoubtedly had partly contributed to the inferior LDL-C reduction seen in group A compared to the better tolerated strategy in group AE.

In other words, the significantly lower frequency of DR/DC events certainly had led to a more sustained and relatively superior LDL-C reduction in the group AE compared to group A. Collectively, these distinct differences in tolerability and the resultant overall efficacy were associated with a significantly lower 1-year-MACE (with RRR of 25.7%), and a trend towards fewer MI and heart failure events in the AE group.

**Fig. 4:**
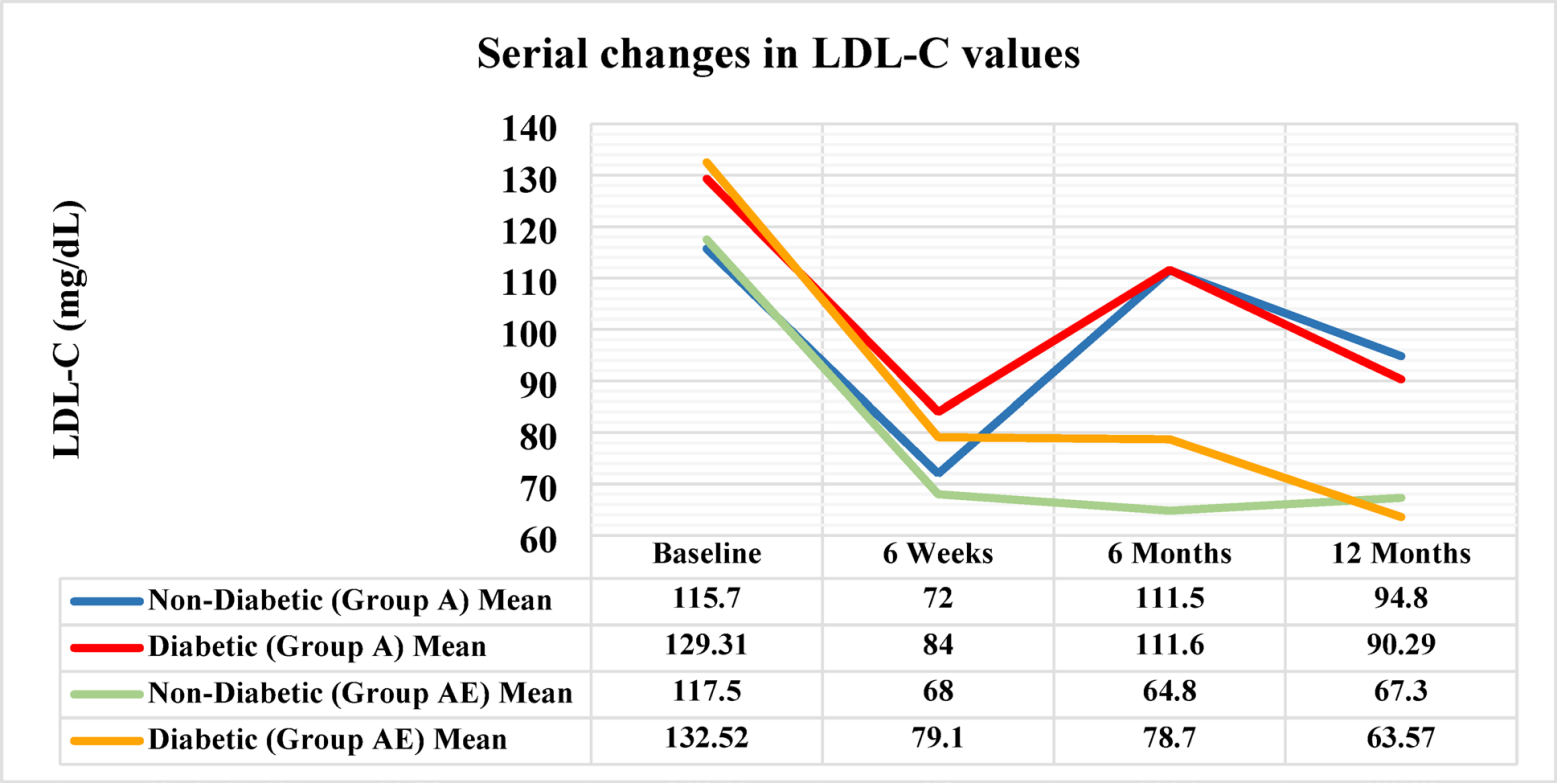
Trends in LDL-C over time stratified by diabetes status and treatment allocation. The study timepoints plotted on the X-Axis and LDL-C level represented on the Y-axis in mg/dL. Each arm is illustrated in the corresponding line

We conducted a dedicated subgroup analysis to recognize the impact of diabetes status on the performance of the studied treatment strategies (Fig.4). Although the diabetic patients had significantly higher baseline LDL-C compared to non-diabetics, the percent reduction of LDL-C demonstrated a more sustained and consistent reduction in group AE compared to group A, as demonstrated by the serial and the final assessments. However, this was not translated into a significant difference in rate of MACE occurrence between treatment arms when stratified by diabetes status as confirmed by the layered contingency tables as well as the multivariate logistic regression. Such a finding may be due to the relatively short follow up period, while the superior LDL-C reduction may require a longer follow-up period to witness a divergence in MACE rates. Worth mentioning that among the 4933 diabetic patients recruited in the IMPROVE-IT trial, the ezetimibe plus statins arm outperformed statins only arm in LDL-C reduction, (mean LDL-C 89 vs. 97 mg/dl, *P* < 0.001), yet without a significant difference in MACE up to 4-years [30, 33].

In summary, although high-intensity statins therapy is the backbone LDL-C lowering for patients with ASCVD, it scarcely alone achieves the target levels. Amid their high cost, the utilization of the majority of non-statin therapies is quite restricted with the exception of Ezetimibe, which is a well-tolerated very affordable agent. Furthermore, the benefits of LDL-C lowering needs consistent adherence and compliance, underscoring the importance to consider the tolerability and affordability of the prescribed therapy as critical as the intensity.

Hereby, our findings provide supportive evidence that a starting regimen with atorvastatin 40 mg plus ezetimibe 10 mg can lead to superior LDL-C reduction with significantly improved safety and tolerability compared to starting atorvastatin 80 mg. Notably, our results do not encourage to reduce the dose for patients well-tolerating atorvastatin 80 mg, as that was not the tested hypothesis in the study and these patients were excluded from recruitment.

## Limitations

This study had certain limitations to acknowledge. Ideally, this study would have been designed with double blinding to eliminate potential bias in SAMS reporting, however, blinding was impeded by several logistic and administrative limitations. Nevertheless, dependence on several objective parameters such as LDL-C levels on serial assessment and clearly defined guiding rules with CPK levels to mandate DC and/or DR, were among the efforts to minimize subjectivity in evaluating treatment arms. Also, ensuring to have a single-pill regimen in both treatment arms aimed to eliminate differences of patients’ compliance if an arm was receiving more than 1 pill. Another limitation was the lack of utilization of injectable LDL-C lowering agents, such as PCSK9i or SiRNA, yet this was because the majority of participants were recruited from university and general hospitals where these agents are not supported by the medic-aid systems. Nevertheless, such a limitation makes the study very representative for the real-world data and the preponderance of everyday practice. Considering the clinical endpoint by the composite of MACE was essentially selected in the study design to attain an adequate study power, as the expected event rates for individual components would require a much larger number of study participants and a longer period of follow-up to achieve the thresholds for between-groups statistical significance.

## Conclusion

Compared to atorvastatin 80 mg, the combination of atorvastatin 40 mg plus ezetimibe 10 mg can represent a compelling starting alternative for ACS patients. Because of better consistency, enhanced tolerability, and fewer discontinuations, the combination therapy may deliver comparable or better clinical outcomes with significantly fewer adverse events.

## Data Availability

No datasets were generated or analysed during the current study.

## Abbreviations

ACC: American College of Cardiology
ACS: Acute coronary syndrome
ALT: Alanine aminotransferase
AMI: Acute myocardial infarction
ASCVD: Athero–Sclerotic CardioVascular Disease
BMI: Body mass index
CAD: Coronary artery disease
CBC: Complete Blood Count
CI: Confidence interval
CK: Creatine kinase
CTT: Cholesterol Treatment Trialist
CVA: Cerbro–Vascular accident
DC: Dose reductions
DR: Dis-continuation
ECG: Electrocardiogram
ESC: European Society of Cardiology
GOULD: Getting to an ImprOved Understanding of Low-Density Lipoprotein–Cholesterol and Dyslipidemia Management
GRACE: Global Registry of Acute Coronary Events
HDL-C: High-density lipoprotein cholesterol
hs-CRP: High-sensitivity C-reactive protein
IMPROVE-IT: Improved Reductions of Outcomes: Vytorin Efficacy International Trial
LDL-C: Low Density Lipoprotein-Cholesterol
MACE: Major Adverse Cardiac Events
NHANES: National Health and Nutrition Examination Survey
NLA: National Lipid Association
NPC1L1: Niemann-Pick C1-Like-1
NSTE-ACS: Non-ST segment elevation acute coronary syndrome
NSTEMI: Non-ST segment elevation myocardial infarction
OR: Odds ratio
PCI: Percutaneous coronary intervention
PCSK9i: Proprotein Convertase Subtilisin/Kexin Type 9 Inhibitor
RRR: Relative risk reduction
SAMS: Statins-associated muscle symptoms
SiRNA: Small Interfering RNA
STEMI: ST-segment elevation myocardial infarction
TC: Total cholesterol
TGs: Triglycerides
TNT: Treatment to New Targets
UA: Unstable angina
